# Effect of percutaneous electrical stimulation with high-frequency alternating currents at 30 kHz on the sensory-motor system

**DOI:** 10.3389/fnins.2023.1048986

**Published:** 2023-02-09

**Authors:** David Martín-Caro Álvarez, Diego Serrano-Muñoz, Juan José Fernández-Pérez, Julio Gómez-Soriano, Juan Avendaño-Coy

**Affiliations:** Toledo Physiotherapy Research Group (GIFTO), Faculty of Physiotherapy and Nursing of Toledo, Universidad de Castilla-La Mancha, Toledo, Spain

**Keywords:** nerve block, high-frequency alternating currents, percutaneous electric stimulation, peripheral nerve, sensorimotor function

## Abstract

**Background:**

Unmodulated high-frequency alternating currents (HFAC) are employed for producing peripheral nerves block. HFAC have been applied in humans with frequencies up to 20 kHz, whether transcutaneously, percutaneously, or *via* surgically-implanted electrodes. The aim of this study was to assess the effect of percutaneous HFAC, applied with ultrasound-guided needles at 30 kHz, on the sensory-motor nerve conduction of healthy volunteers.

**Methods:**

A parallel, double-blind, randomized clinical trial with a placebo control was conducted. Percutaneous HFAC at 30 kHz or sham stimulation was applied *via* ultrasound-guided needles in 48 healthy volunteers (*n* = 24 in each group) for 20 min. The assessed outcome variables were pressure pain threshold (PPT), mechanical detection threshold (MDT), maximal finger flexion strength (MFFS), antidromic sensory nerve action potential (SNAP), hand temperature, and subjective sensations by the participants. The measurements were recorded pre-intervention, during the stimulation (at 15 min), immediately post-intervention (at 20 min), and 15 min after the end of treatment.

**Results:**

The PPT increased in the active group compared with sham stimulation, both during the intervention [14.7%; 95% confidence interval (CI): 4.4–25.0], immediately post-intervention (16.9%; 95% CI: −7.2–26.5), and 15 min after the end of the stimulation (14.3%; 95% CI: 4.4–24.3) (*p* < 0.01). The proportion of participants who reported feelings of numbness and heaviness was significantly higher in the active group (46 and 50%, respectively) than in the sham group (8 and 18%, respectively) (*p* < 0.05). No intergroup differences were observed in the remaining outcome variables. No unexpected adverse effects derived from the electrical stimulation were reported.

**Conclusion:**

Percutaneous stimulation with HFAC at 30 kHz applied to the median nerve increased the PPT and subjective perception of numbness and heaviness. Future research should evaluate its potential therapeutic effect in people with pain.

**Clinical trial registration:**

https://clinicaltrials.gov/ct2/show/NCT04884932, identifier NCT04884932.

## 1. Introduction

High-frequency alternating currents (HFAC) stimulation at > 1 kHz has shown to produce peripheral nerve block that is quickly reversible and does not cause damage to the nerve (Kilgore and Bhadra, 2004; Tai et al., 2004; Bhadra and Kilgore, 2005; Bhadra et al., 2006, 2007; Ackermann et al., 2011; Ling et al., 2019; Ray et al., 2021). Preclinical trials employing HFAC have reported effective nerve block applying a wide range of frequencies between 1 and 300 kHz (Wang et al., 2008; Zhao et al., 2015; Avendaño-Coy et al., 2017; Avendano-Coy et al., 2018; Ray et al., 2021). Research in primates, whose median nerve is the most similar to that of humans, showed that the minimum frequency to achieve complete conduction blockade was 20 kHz (Ackermann et al., 2011). However, there is no solid evidence about the optimum frequency to elicit nerve block in humans. To date, studies have applied frequencies of 5, 10, and 20 kHz transcutaneously and all have reported a reduction in voluntary strength (Kim et al., 2018; Serrano-Muñoz et al., 2018, 2020) and increases in both mechanical and thermal somatosensory pain thresholds as well as greater tactile sensitivity (Avendaño-Coy et al., 2017; Kim et al., 2018), with an effect that lasts up to 10 min after finalizing the stimulation (Serrano-Muñoz et al., 2018, 2020; Springer et al., 2018). However, only a pilot trial conducted by our research group has employed HFAC at frequencies above 20 kHz. Specifically, transcutaneous HFAC at 30 kHz was applied to the median nerve, a procedure that proved to be safe and resulted in a decrease in the maximum handgrip strength (Serrano-Muñoz et al., 2022).

The main limitation of applying HFAC transcutaneously is the distance between the electrode and the nerve. Research in animals has shown that the longer the distance between them, the higher the intensity required to reach nerve block (Bhadra and Kilgore, 2005). Ackermann et al. (2009) employed implanted electrodes in rats and determined that, in order to lower the intensity that attained nerve block, the most effective distance from the electrode to the axon was 1–2 mm. Clinical trials in humans have applied HFAC *via* electrodes implanted at the intrafascicular level (Dowden et al., 2010) and also with ring electrodes placed around the nerve (Rubinstein et al., 2003; Soin et al., 2015). Although both procedures have shown positive results, they entail risks derived from additional interventions to replace electrodes (Finch et al., 2019). The ultrasound-guided percutaneous application of currents can be considered a minimally invasive procedure (Boyce et al., 2020) that allows reducing the distance between the electrode and nerve (Williamson and Andrews, 2005) and has proven to be a safe method with minimum associated risks (Eldabe et al., 2016; No author list, 2019). Our research group observed in a pilot study that the percutaneous ultrasound-guided application of 20 kHz HFAC to the median nerve was a feasible, safe procedure with minimal risks and with a potential effect on the pressure pain threshold (PPT) (No author list, 2019). The outcomes of another recent study that applied ultrasound-guided HFAC at 10 and 20 kHz *via* needle electrodes showed that this safe procedure produced a decrease in the maximum handgrip strength using 20 kHz, but not 10 kHz, without any changes being observed in any somatosensory threshold (Álvarez et al., 2022).

The main aim of the current study was to assess the effect of HFAC, applied percutaneously to the median nerve at 30 kHz, on the somatosensory and motor activity compared to sham percutaneous electric stimulation. The secondary objectives were the evaluation of adverse effects, hand temperature, and subjective perception of the participants during and after the intervention.

## 2. Materials and methods

### 2.1. Design

A parallel randomized clinical trial was conducted in 48 healthy volunteers with a double-blind placebo control. Prior to the beginning of the trial, all participants provided informed written consent that had been previously approved by the local ethics committee (ref. 441; 11/11/2019) and registered at www.ClinicalTrials.gov (NCT04884932).

A simple balanced randomization was performed on the website www.randomizer.org. An external researcher randomly assigned the participants to two intervention groups (30 kHz and sham stimulation). This investigator assigned one group, or another based on the code assigned on the randomization sheet. Both the subjects and evaluators were blinded to the intervention group, which was kept in a closed envelope throughout the study period so that only the researcher delivering the intervention was aware of the participants’ allocation.

The intervention duration was 20 min. The measurements were recorded pre-intervention, during the stimulation (at 15 min), immediately post-intervention (at 20 min), and 15 min after the end of treatment (Avendaño-Coy et al., 2017). However, the sensory nerve action potential (SNAP) and maximal finger flexion strength (MFFS) of the index could not be recorded during the intervention due to the location of the needle and discomfort during muscle contraction.

### 2.2. Subjects

Healthy volunteers were recruited between 18 and 40 years of age, without pathologies of the central or peripheral nervous system, nickel allergy, or intolerance to the percutaneous application of electric currents. The criteria for exclusion were: previous surgery or osteosynthesis material in the upper limb where the intervention was to be applied, epilepsy, infectious disease, neuromuscular disease, heart condition, diabetes, cancer, pacemaker or any other implanted electric device, pregnancy, tattoo or any skin alteration preventing the application of currents, and substance or drugs use (anti-clotting medication, thrombolytic agents, pain killers, corticoids, antidepressants, antiepileptic drugs) during the study or in the seven previous days.

### 2.3. Intervention

The interventions were performed on the non-dominant upper limb of the participants in the supine decubitus position. Antiseptic and disinfection treatment with 2% alcohol-based chlorhexidine was applied on the skin at the intervention area. A bilateral ultrasound-guided percutaneous approach was performed with a Samsung HS50 ultrasound device (Samsung healthcare; Seoul, South Korea) fitted with a 12 MHz linear probe, with a short-axis approach to the median nerve on the anterior aspect of the middle third of the forearm, placing two acupuncture needles 0.30 mm x 40 mm (Agupunt^®^; Barcelona, Spain), one on each side of the nerve close to the epineurium of the median nerve (1 mm). The average depth of the needle introduced into the tissue was approximately 3 cm.

A Myomed 932 (Enraf-Nonius; Delft, The Netherlands) device delivered the current with each electrode connected to the needle through a clasp. The same stimulator was employed in both the active and sham interventions, which were delivered in a research laboratory under attenuated-noise conditions and room temperature of 21–25°C.

#### 2.3.1. 30 kHz stimulation

The electric current in the active group was unmodulated, biphasic, with symmetric sinusoid waveform. The frequency was 30 kHz and the stimulation intensity was progressively increased until reaching a feeling of “strong but comfortable” tingling just below the motor threshold (Claydon et al., 2013), for which the intensity was raised until a minimally visible contraction was observed and then slightly lowered below the motor threshold. Due to habituation to the stimulus, the intensity was adjusted every 2 min and raised if the perception of the current by the participants decreased (Serrano-Muñoz et al., 2017).

#### 2.3.2. Sham stimulation

**FIGURE 1:**
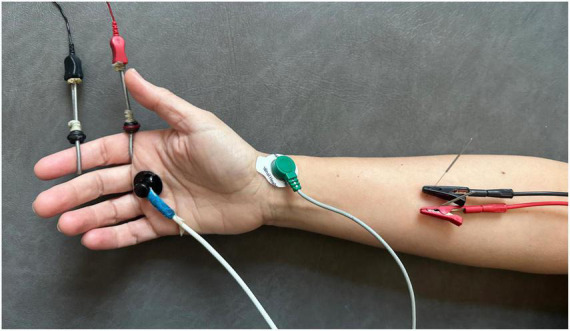
Procedure of application of percutaneous electrical stimulation with high-frequency alternating currents (HFAC).

The sham stimulation was applied with the same device, needle placement, and parameters as in the 30 kHz intervention, except for the current intensity that was initially adjusted up to the sensory threshold and, once the patient perceived a tingling sensation for a few seconds, the intensity was gradually decreased to and maintained at 0 mA throughout the session (Figure 1).

### 2.4. Outcome measures

#### 2.4.1. Somatic sensitivity: Pressure pain threshold

The main outcome variable was the PPT. A digital algometer with 0.1 N force increments and a circular tip of 1 cm in diameter (Wagner Instruments, model FDIX; Greenwich, USA) was employed to measure this outcome on the palmar aspect of the trapeziometacarpal joint. The pressure was increased at a rate of approximately 5 N/s (Chesterton et al., 2002). Three measurements were recorded with intervals of 10 s between them (Nussbaum and Downes, 1998) and the PPT value (in N) was calculated as the average of the three measurements (Fischer, 1987; Antonaci et al., 1992, 1998).

#### 2.4.2. Somatic sensitivity: Mechanical detection threshold

The mechanical detection threshold (MDT) was measured on the palmar aspect of the hand, in an area of 1 cm^2^ proximal to the head of the second metacarpal in the thenar eminence, using modified Von Frey filaments of 0.4 mm of diameter (OptiHair2, MARSTOCKnervtest; Marburg, Germany) exerting forces of 0.25, 0.5, 1, 2, 4, 8, 16, 32, 64, 128, 256, and 512 mN (Ristić et al., 2008). Seven stimuli were applied with the filament and the threshold was determined when at least four of them were perceived (Nilsson and Schouenborg, 1999).

#### 2.4.3. Maximal flexion strength of the index finger

Motor activity was assessed *via* the MFFS, which was measured with the subjects in the supine position and their hand in pronation pressing with the distal phalanx of the index finger on a MicroFet 2 TM digital hand dynamometer (Hoggan Scientific, LLC; Utah, USA). This method has shown a good intra- and inter-rater reliability (Mentiplay et al., 2015). Three measurements were taken with a contraction time of 3 s and rest intervals of 5 s, and the MFFS value (in kg) was estimated as the mean of the three measurements (Shiratori et al., 2014).

#### 2.4.4. Sensory nerve action potential (SNAP)

The SNAP of the median nerve was recorded to evaluate the effect on peripheral nerve conduction (Valls-Sole et al., 2016). Nerve stimulation was performed on the medial aspect of the arm, 4 cm cranial to the medial epicondyle, by means of a transcutaneous bipolar electrode with a fixed distance between electrodes of 1 cm, placing the cathode about 40 cm from the recording electrode. Two ring electrodes were placed around the index finger to record the potential, one on the proximal interphalangeal joint and another on the distal interphalangeal joint, with the grounding electrode on the radial side of the wrist joint (Letz and Gerr, 1994; Walsh et al., 1998). Supramaximal stimuli were applied with a pulse width of 1,000 μs and a frequency of 1 Hz *via* a constant-current stimulator (Digitimer LTD, model DS7A; Letchworth Garden City, United Kingdom), an analogic/digital data acquisition card (Cambridge Electronic Devices; Cambridge, United Kingdom), and an amplifier (model ETH-256, iWorxs; Dover, USA) with a 3-Hz high-pass filter and 2,000-Hz low pass filter. The recorded variables were the mean value of ten recordings of the SNAP amplitude and onset latency of the potential. At the baseline, the mean of two SNAP values with a two-minute interval was obtained to analyze the stability of the potential by calculating the intraclass correlation coefficient of both measurements.

### 2.5. Hand temperature

Hand temperature was recorded using a temperature monitor (model DRT4, Moor Instruments brand; Devon, United Kingdom), with the recording sensor placed distal to the intervention, on the palmar side of the head of the first metacarpal (Avendaño-Coy et al., 2017). Additionally, the ambient room temperature was registered.

### 2.6. Adverse effects and subjective sensations

A questionnaire was developed *ad hoc* and completed at the end of the intervention to evaluate adverse effects and the subjective perception of the participants. Nine items with a “Yes/No” answer were included to assess pain, inflammation, reddening, cold feeling, numbness, strength loss, heaviness, and tingling in the hand and intervention areas. A numerical scale ranging from 0 to 10 was also employed to evaluate discomfort and/or pain caused by the intervention, where 0 corresponded to “not at all” and 10 to “the maximum conceivable.” In addition, participants were asked to report whether they had perceived any of the above-mentioned effects or sensations in the intervention area within the 24 h following the intervention.

### 2.7. Blinding assessment

The successful blinding of both the participants and evaluator was assessed after the intervention ended (Bang et al., 2010) using a closed-ended question: “What type of treatment do you think you received?” with five response options: (1) “I strongly believe that I received an experimental treatment”; (2) “I somewhat believe that I received an experimental treatment”; (3) “I strongly believe that I received a placebo”; (4) “I somewhat believe that I received a placebo”; and (5) “Do not know, do not answer.”

### 2.8. Statistical analysis

The Epidat 3.1 software was employed to calculate the sample size based on a previous pilot test (No author list, 2019). For an expected between-group mean difference (MD) in the PPT of 10.3 N/cm^2^, with a standard deviation (SD) of 11.3 N/cm^2^ and 9.9 N/cm^2^ in the experimental and control groups, respectively, and considering a type I error (α) of 0.05 and a power of 85%, the sample size was estimated to be 20 subjects per group (*n* = 20). To compensate for possible dropouts, a supplementary 20% was added to the sample, yielding a total of *n* = 24 participants per group.

For the comparison of baseline characteristics between groups, a descriptive analysis and inferential statistics for basal demographic variables was performed for independent groups (parametric or non-parametric depending on the variable). For the intragroup comparisons, a repeated-measures analysis of variance (ANOVA) with a Bonferroni *post hoc* test was conducted for the following outcome variables: PPT, MDT, MFFS, hand temperature, and SNAP. For those variables violating sphericity, the Greenhouse-Geisser correction was employed. For between-group comparisons, changes in the mentioned variables with respect to the baseline were assessed using the Student’s *t*-test for independent samples. Prior to the analysis, the analyzed outcome variables were normalized in percentages with respect to the baseline.

A Chi-squared test was performed to analyze adverse effects and a Student’s *t*-test for independent samples was used to evaluate unpleasantness and pain during the intervention. A value of *p* < 0.05 was considered statistically significant. The IBM SPSS Statistic 24.0 for Mac was used for the statistical analyses.

## 3. Results

**FIGURE 2:**
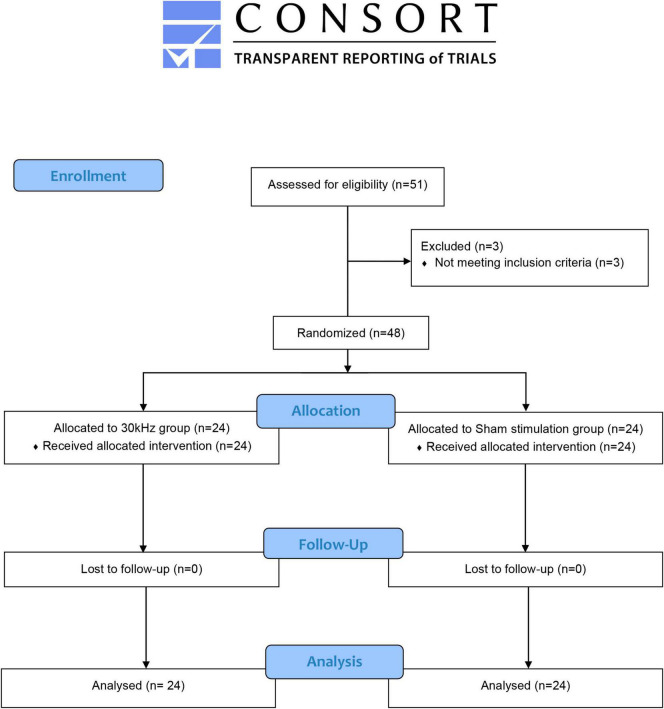
Consort flow diagram.

Forty-eight participants completed the study and were included in the analysis (Figure 2). No differences in demographic variables were found between groups at the baseline (see Table 1). The mean current intensity applied was 4.0 mA (SD 2.8) at the beginning of the intervention and 19.0 mA at the end (SD 10.8). Taking into account that the average depth of the needle introduced into the tissue was 3 cm, the electrical current density was calculated to be around 14.3 and 67.8 mA/cm^2^ at the beginning and end of the intervention, respectively. Supplementary Table shows the non-normalized values of outcome variables recorded at the four assessment points.

**TABLE 1:** Demographic characteristics of participants at the baseline.

Outcomes	All participants (*n* = 48)	30 kHz Group (*n* = 24)	Sham Group (*n* = 24)	Between-group differences (*p*-value)
Age (years) Mean (SD)	21.0 (2.5)	20.5 (1.7)	21.5 (3.0)	(*p* = 0.20)**^a^**
Gender Men, n (%)	24 (50.0%)	11 (45.8%)	13 (54.2%)	(*p* = 0.56)**^b^**
Weight (kg) Mean (SD)	65.8 (11.0)	66.6 (10.1)	64.9 (12.0)	(*p* = 0.59)**^a^**
Height (m) Mean (SD)	1.70 (0.08)	1.71 (0.08)	1.70 (0.09)	(*p* = 0.53)**^a^**
Body *mass index* (kg/m^2^) Mean (SD)	22.5 (2.6)	22.7 (2.7)	22.4 (2.5)	(*p* = 0.65)**^a^**
Non-dominant hand Left, n (%)	45 (93.8%)	21 (87.5%)	24 (100.0%)	(*p* = 0.23)**^c^**

Statistical test: a) Student’s *t*-test for independent samples, b) Pearson’s chi-squared test, c) Fisher’s exact test.

### 3.1. Pressure pain threshold and mechanical detection threshold

Differences in the PPT were observed in the “time” factor (*F* = 5.6; *p* = 0.002) and the intersection “time-intervention” (*F* = 5.2; *p* = 0.003). A clinically relevant increase in the PPT was found in the 30 kHz group during the intervention compared to the baseline, in contrast with the sham group, where a significant decrease was observed immediately post-intervention and 15 min after its finalization (see Table 2). In the intergroup comparison of changes over time, differences in the intervention effect on the PPT were observed during the intervention (*t* = 2.9; *p* = 0.006), immediately post-intervention (*t* = 3.5 *p* = 0.001), and 15 min after the end of the stimulation (*t* = 2.9; *p* = 0.006), with a greater increase in the 30 kHz group compared to the sham stimulation group (Table 3). In contrast, no changes in the MDT outcome were observed in the intragroup (Table 2) or intergroup comparisons (Table 3) at any time point.

**TABLE 2:** Intragroup comparison in outcome variables from baseline.

	Intragroup comparison from baseline					
	**Sham group**			**30 kHz group**		
**Outcomes% Mean (CI95%)**	**During minus Pre**	**Post intervention minus Pre**	**Post 15 min intervention minus Pre**	**During minus Pre**	**Post intervention minus Pre**	**Post 15 min intervention minus Pre**
Pain pressure threshold (%)	−4.3 (−14.3–5.6)	−11.6** (−**21.0**–−**2.3)**	−13.4** (−**23.0**–−**3.8)**	10.4* (0.4–***20.3)***	5.2 (−4.1–14.6)	0.9 (−8.7–10.6)
Mechanical detection threshold (%)	32.5 (−32.3–97.3)	−7.5 (−49.8–34.8)	5.0 (−54.5–64.5)	46.3 (−18.5–111.2)	−4.9 (−47.2–37.4)	−9.7 (−69.2–49.7)
Strength (%)	NA	−11.0** (−**19.1**–−**3.0)**	−2.8 (−11.0–5.3)	NA	−14.7** (−**22.7**–−**6.6)**	−8.8* (−**17.0**–−**0.7)**
Nerve conduction velocity (%)	NA	−5.9** (−**9.2**–−**2.5)**	−9.6** (−**16.6**–−**2.5)**	NA	−5.5** (−**8.9**–−**2.2)**	−7.9* (−**15.0**–−**0.9)**
SNAP Amplitude (%)	NA	34.6 (−16.2–85.5)	24.5 (−24.0–73.0)	NA	45.8 (−5.0–96.7)	49.7* (1.2–**98.2)**
Hand temperature (%)	−1.6 (−4.6–1.3)	−2.2 (−5.6–1.2)	−2.7 (−6.6–1.3)	−3.6** (−**6.6**– −**0.7)**	−4.5** (−**7.8**–−**1.1)**	−5.1** (−**9.0**–−**1.1)**

Bold values denote statistical significance: (*) *p* < 0.05 level (**); *p* < 0.01 level. Pre, Immediately pre-intervention; During, During the intervention at 15 min; Post 0, Immediately post-intervention; Post 15, at 15 min after the end of the stimulation; NA, Not applicable; SNAP, Sensory nerve action potential.

**TABLE 3:** Intergroup comparison of changes from baseline.

	Intergroup comparison of changes from baseline		
**Outcomes**	**Change 30 kHz minus Change Sham**		
**% Mean (CI95%)**	**During intervention**	**Post** **Intervention**	**Post 15 intervention**
Pressure pain threshold (%)	14.7** (4.4–**25.0)**	16.9** (7.2–**26.5)**	14.3** (4.4–**24.3)**
Mechanical detection threshold (%)	13.8 (−53.1–33.3)	2.6 (−41.0–21.7)	−14.7 (−76.1–30.5)
Strength (%)	NA	−3.6 (−12.8–5.6)	−6.0 (−15.3–3.3)
SNAP Amplitude (%)	NA	11.2 (−47.0–69.4)	25.2 (−30.3–80.7)
Nerve velocity conduction (%)	NA	0.3 (−3.5–4.1)	1.6 (−6.5–9.7)
Hand temperature (%)	−2.0 (−5.1–1.1)	−2.2 (−5.7–1.3)	2.4 (−6.5–1.7)

Bold values denote statistical significance: (**) *p* < 0.01 level; NA, Not applicable; SNAP, Sensory nerve action potential.

### 3.2. Maximal flexion strength of the index finger

Significant differences in the MFFS were found in the “time” factor (*F* = 17.6; *p* < 0.0001) but not in the “time-intervention” intersection (*F* = 1.0; *p* = 0.38). The MFFS decreased in the 30 kHz group immediately post-intervention, similarly to the sham group (see Table 2). However, no intergroup differences in the changes in MFFS were observed at any time point (see Table 3).

### 3.3. Sensory nerve action potential and temperature

The SNAP was stable at the baseline, with an intraclass correlation coefficient of 0.97 (0.94–0.98) between the two recorded values. Significant differences were recorded in the nerve conduction velocity in the “time” factor (*F* = 14.1; *p* < 0.001), but not in the intersection “time-intervention” (*F* = 0.1; *p* = 0.87). The conduction velocity decreased in both groups immediately post-intervention and 15 min after the end of stimulation (Table 2). Significant differences in the potential amplitude were observed in the “time” factor (*F* = 7.0; *p* = 0.001) but not in the “time-intervention” intersection (*F* = 0.6; *p* = 0.49). An increase in the potential amplitude with respect to the baseline was found only in the 30 kHz group both during the intervention and 15 min after the commencement of the stimulation (Table 2). The intergroup comparison of the intervention effect did not reveal differences in the nerve conduction velocity or the potential amplitude (Table 3).

Significant changes in hand temperature were detected in the “time” factor (*F* = 12.7; *p* < 0.001) but not in the “time-intervention” intersection (*F* = 1.4; *p* = 0.26). Hand temperature significantly decreased in the 30 kHz group during the stimulation, immediately post-treatment, and 15 min after the end of the intervention, in contrast with the sham group, where no changes were observed over time (Table 2). No intergroup differences in the effect on hand temperature were found at any time point (Table 3).

### 3.4. Subjective variables

Table 4 shows the outcomes of the subjective perceptions by the participants. Statistically significant differences between groups were only registered in the sensations of numbness (χ^2^:8.6, *p* = 0.008) and heaviness (χ^2^: 6.0, *p* = 0.03). No unexpected adverse effects were recorded or reported by the participants other than those derived from puncturing.

**TABLE 4:** Subjective perception by the participants.

*n* (%)	30 kHz (*n* = 24)	Sham stimulation (*n* = 24)	*P*-value ^(a)(b)^
Pain	2 (8.3%)	0 (0%)	*p* = 0.49
Numbness	11 (45.8%)	2 (8.3%)	***p* = 0.008**
Cold	6 (25%)	7 (29.2%)	*p* = 0.75
Loss of strength	9 (37.5%)	3 (12.5%)	*p* = 0.09
Heaviness	12 (50%)	4 (16.7%)	***p* = 0.03**
Tingling	7 (29.2%)	3 (12.5%)	*p* = 0.29
Inflammation	0 (0%)	0 (0%)	NA
Erythema	3 (12.5%)	2 (8.3%)	*p* = 1.0
Heat	0 (0%)	0 (0%)	*NA*
Blood pressure drop	4 (16.7%)	3 (12.5%)	p1.0
**Mean (SD)**	**30 kHz (*n* = 24)**	**Sham stimulation (*n* = 24)**	***P*-value^(c)^**
Pain (0–10)	4.2 (2.0)	3.5 (2.3)	*p* = 0.32
Unpleasantness (0–10)	4.8 (1.8)	4.2 (2.3)	*p* = 0.33

^(a)^ Pearson’s chi-squared test, ^(b)^ Fisher’s exact test (cells with *n* < 5), ^(c)^ Student’s *t*-test for independent samples; *NA = not applicable.

### 3.5. Blinding assessment

Table 5 displays the outcomes of the blinding of the participants and evaluator. The overall analysis *via* the James’ index (James et al., 1996) determined the correct blinding of the participants and the lack of blinding of the evaluator. The blinding analysis by intervention group using the Bang’s index (Bang et al., 2004, 2010) revealed a lack of blinding in the active group of both the participants and assessor, who correctly guessed the allocation in 46 and 48% of cases, respectively. In the sham group, 44% of participants believed they were assigned to the active group (opposite guess) and the evaluator guessed the allocation in 46% of cases, which indicates a lack of blinding.

**TABLE 5:** Statistical analysis of blinding assessment.

Participants blinding				
**Methods**	**Index**	***p*-value**	**95% Confidence interval**	**Conclusion**
James’ BI	0.47	0.307	0.38 to 0.56	Blinded
Bang’s BI-Active/2 × 5	0.46	*p* < 0.001	0.27 to 0.64	Unblinded
Bang’s BI-Placebo/2 × 5	−0.44	1	−0.65 to −0.22	Opposite guess*
**Assessor blinding**				
**Methods**	**Index**	***p*-value**	**95% Confidence interval**	**Conclusion**
James’s BI	0.31	*p* < 0.001	0.20 to 0.42	Unblinded
Bang’s BI-Active/2 × 5	0.48	*p* < 0.001	0.25 to 0.71	Unblinded
Bang’s BI-Placebo/2 × 5	0.46	*p* < 0.001	0.24 to 0.68	Unblinded

*Wishful thinking, participants tend to think they are allocated to the active group even if they are not. BI, blinding index.

## 4. Discussion

To our knowledge, this is the first clinical trial that applied percutaneous ultrasound-guided HFAC at frequencies of 30 kHz. The results showed increases in the main outcome variable PPT compared to placebo stimulation of 14.7% during the intervention, 16.9% at the end of the stimulation, and 14.3% at 15 min after the end of the current application. No differences between groups were observed in the remaining assessed variables.

The data obtained in this study showed increases in the PPT at all evaluation time points, revealing the selective effect of 30 kHz HFAC stimulation that mainly affects Aδ-fibers. These outcomes could be considered clinically relevant according to the studies by Chesterton et al. (2002, 2003), which estimated that changes greater than 10 N/cm^2^ versus the baseline were clinically relevant. There was a decrease in PPT in the placebo stimulation group at the end of the intervention and 15 min after its finalization, which was likely due to the sensitization and irritation of the cutaneous nociceptors produced by successive evaluations, as reported by Sjölund and Persson (2007). Serrano-Muñoz et al. (2020) applied transcutaneous HFAC at 20 kHz and did not report changes in the PPT. Similarly, a former trial by our research group (Álvarez et al., 2022) that applied ultrasound-guided percutaneous HFAC at 10 kHz and 20 kHz did not find changes in the PPT versus sham stimulation. However, the latter trial reported a superior effect with 20 kHz on muscle contraction (Aα-fibers), both in reducing muscle strength and increasing myotonometry, than that obtained with placebo (Álvarez et al., 2022).

The differences in the effect achieved in humans depending on the employed frequency are in agreement with the evidence observed in preclinical animal testing and computer simulation studies. Different research suggests that HFAC stimulation causes the selective blockade of specific nerve fibers depending on the frequency and intensity applied (Tai et al., 2005a,b; Bhadra et al., 2007; Dowden et al., 2010; Waataja et al., 2011; Sun and Liu, 2013; Avendano-Coy et al., 2018). Joseph and Butera (2011) reported that the blocking threshold of large-diameter fibers (Aα and Aβ) was lower than that of smaller-diameter fibers (Aδ and C) with frequencies below 30 kHz. On the contrary, when the frequency was equal to or above 30 kHz, the blocking threshold of Aδ- and C-fibers was lower. Human research with frequencies higher than 30 kHz is necessary to confirm this relationship between the current frequency and the selective block of small-diameter fibers.

Human trials evaluating HFAC at frequencies below 30 kHz have shown an effect on the sensory and motor conduction functions, which involve large-diameter fibers (Aα and Aβ). The studies by Avendaño-Coy et al., 2017 and Kim et al. (2018), which applied HFAC at 5 kHz and 10 kHz, respectively, reported an effect on the MDT, which is related to Aβ-fibers. However, the present trial did not reveal changes in the MDT, likely because the employed frequency (30 kHz) presents a higher blockade threshold for this type of fibers. No intergroup differences in the nerve conduction velocity or the SNAP amplitude were observed. These variables depend on large-caliber fibers (Aα and Aβ) (Drenthen et al., 2008; Gavanozi et al., 2020), as is the case for the MDT, on which HFAC at 30 kHz appears to have no effect. The same effect was observed in the component involved in motor conduction (fibers Aα) since a decrease in MFFS was found in both the active and sham groups without significant differences between them. These outcomes are in contrast to those obtained with transcutaneous and percutaneous stimulations at 10 kHz and 20 kHz, where differences in the motor component were recorded compared to the sham group (Álvarez et al., 2022; Serrano-Muñoz et al., 2018, 2020).

Despite the lack of differences between groups, intragroup changes in the conduction velocity and amplitude of the SNAP were observed, as well as in hand temperature. Decreases in nerve conduction velocity and hand temperature were observed during the current application, immediately post-stimulation, and 15 min. after the intervention, together with an increase in the SNAP amplitude at this measurement point. Aδ- and C-fibers are responsible for temperature afferents (Ackerley and Watkins, 2018), with C-fibers presenting a vegetative sympathetic component (Oaklander, 2016). A direct relationship between body temperature and nerve conduction has been demonstrated (Franssen and Wieneke, 1994; Notermans et al., 1994). Body temperature affects nerve conduction (Tai et al., 2008; Wang et al., 2008), possibly related to potassium channels (Tai et al., 2009), so the descent in hand temperature observed in the active group could be responsible for the decrease in the nerve conduction velocity and changes in the SNAP. The findings of the present study indicate a potential effect of HFAC of 30 kHz on the nerve conduction of slower, thinner fibers (Aδ-fibers and C-fibers), which suggests a partial block, although the mechanism underlying this effect is not defined yet (Neudorfer et al., 2021). Previous studies have shown that a toxin can produce the selective blockade of the autonomic nervous system (Stürzebecher et al., 2010), although there is no evidence that this blockade could be produced using electric currents. To date, it has been postulated that there are no electric currents that alter the thresholds of thermal perception, mediated by C- and Aδ-fibers (Palmer et al., 2004), so the findings of the current study with HFAC could be relevant. Despite the absence of intergroup differences, the decrease in hand temperature in the active group could constitute an important finding of the blocking effect of 30 kHz HFAC on vegetative fibers. However, hand temperature was recorded as a secondary variable in the present study, where the sample size was calculated based on the primary outcome variable PPT. Future studies should be designed to study the effect of HFAC on the autonomic nervous system.

Mild adverse effects inherent to puncturing occurred in a low percentage of participants, with no differences between the active and sham groups. The subjective perceptions of numbness and heaviness were significantly more frequent in the active group, affecting 46 and 50% of the participants, respectively. However, both are expected effects of HFAC application and are in line with the objective changes in the PPT observed. No participants reported a sensation of heat or burning in the electrode area during the HFAC stimulation. This differs from the results reported in some studies with implanted electrodes applying 10 kHz currents (spinal stimulation), in which the tissues close to the implanted electrodes suffer a temperature increase (Zannou et al., 2019a,b).

To date, there are no investigations that compare the percutaneous application of HFAC currents with TENS currents. In the transcutaneous application, Avendaño-Coy et al., 2017, showed how the application of 5 kHz and the application of TENS present significant changes in the reduction of the PPT with respect to the placebo group, without finding differences between the active groups. Despite the fact that in the present study there has also been a decrease in the PPT, the mechanisms of action by which one or the other currents act are different. Since while in the application of TENS currents, its effects are due to the activation of descending inhibitory mechanisms at the central level, in the application of HFAC currents, the nerve blocking effect occurs locally, distal to the stimulation site. There is no consensus on the mechanism of action of the nerve block with HFAC currents. However, in computer simulation models it has been shown that this could be due to differences in the ionic gradient of Na+ and K+, although there is controversy as to whether the blockade would be due to the permanent activation of the K+ channels and the inactivation of the Na+ channels (Avendano-Coy et al., 2018).

The present HFAC stimulation protocol could have potential clinical interest for people with pain given the reduction found in experimental pain. Assessing the effect of this protocol in subjects with clinical pain is necessary to evaluate the potential therapeutic effect of HFAC stimulation at 30 kHz.

In future studies, it will be important to investigate which are the most appropriate HFAC application parameters (frequency, stimulation threshold, application time, and electrode location) for the treatment of pathologies that present pain or hyperexcitability of the second motor neuron.

### 4.1. Study limitations

The evaluation of the SNAP and MFFS outcomes during the application of the intervention was not possible due to the placement of the needle electrodes. Although a double-blind study was designed previously, the analysis of blinding success showed a lack of blinding of the evaluator, both globally and by groups, which could result in a detention bias. Future studies should investigate sham stimulations that allow the successful blinding of assessors. Another limitation of the study is that it was performed in healthy participants, so studies in patients with relevant pathologies are warranted to determine the real therapeutic impact of HFAC.

## 5. Conclusion

The percutaneous ultrasound-guided HFAC applied to the median nerve at a frequency of 30 kHz produced an increase in the PPT and greater subjective perceptions of numbness and heaviness compared to sham stimulation. This intervention, which proved to be a safe procedure with minor risks inherent to the puncture, could have a potential clinical application in patients with pain, so future studies should investigate the effect of this intervention in this population.

## Data availability statement

The datasets presented in this study can be found in online repositories. The names of the repository/repositories and accession number(s) can be found below: Original datasets are available in Zenodo repository at doi: https://doi.org/10.5281/zenodo.6637842.

## Ethics statement

The studies involving human participants were reviewed and approved by Ethics Committee of the “Complejo Hospitalario de Toledo” approved this study and warranted it was conducted in accordance with the Declaration of Helsinki (number 441; 11/11/2019). The patients/participants provided their written informed consent to participate in this study.

## Author contributions

DM-CÁ, DS-M, and JF-P contributed to participant recruitment, data collection, and drafting of the manuscript. JA-C, JG-S, and DS-M conceived the study, interpreted the results, and supervised the whole project. All authors read and approved the final manuscript.
