# Supplementary material for: Effect of percutaneous electrical stimulation with high-frequency alternating currents at 30 kHz on the sensory-motor system
Source: Front Neurosci. 2023 Feb 9;17:1048986. doi: 10.3389/fnins.2023.1048986 (PMC9947497; doi:10.3389/fnins.2023.1048986)
Supplement: Supplementary file 1 [file Table_1.DOCX]

| **Outcomes**  Mean (SD) |  | **Supplementary Table: Non-normalized values of outcome variables recorded at the four assessment points.** | | | | | | | | |
| --- | --- | --- | --- | --- | --- | --- | --- | --- | --- | --- |
|  |  | Sham group | | | |  | 30 kHz group | | | |
|  |  | Pre intervention | Dur intervention | Post Intervention | Post 15 min Intervention |  | Pre intervention | Dur intervention | Post Intervention | Post 15 min Intervention |
| Strength (kgs) |  | 5.7 (1.3) | NA | 5.1 (1.1) | 5.6 (1.2) |  | 6.3 (1.3) | NA | 5.4 (1.2) | 5.7 (1.0) |
| Pain Pressure Threshold (N) |  | 50.2 (19.2) | 48.1 (17.5) | 44.4 (16.4) | 43.5 (16.9) |  | 49.7 (19.0) | 54.9 (19.2) | 52.3 (21.5) | 50.2 (21.1) |
| Mechanical Detection Threshold (mN) |  | 0.42 (0.3) | 0.55 (0.5) | 0.38 (0.2) | 0.44(0.4) |  | 0.43 (0.3) | 0.62 (0.5) | 0.41 (0.3) | 0.39 (0.2) |
| SNAP Amplitude (mV) |  | 0.06 (0.08) | NA | 0.09 (0.12) | 0.08 (0.11) |  | 0.05 (0.06) | NA | 0.07 (0.10) | 0.07 (0.10) |
| Nerve Conduction Velocity (m/s) |  | 45.2 (5.5) | NA | 42.5 (6.7) | 40.8 (7.7) |  | 43.6 (7.3) | NA | 41.2 (8.6) | 40.1 (8.8) |
| Hand Temperature (ºC) |  | 27.8 (2.7) | 27.4 (2.9) | 27.2 (2.9) | 27.1 (2.8) |  | 28.3 (2.7) | 27.2 (2.7) | 27.0 (2.7) | 26.8 (2.7) |

NA: Not Applicable. SD: Standard Deviation. SNAP: Sensory nerve action potential. Pre: immediately pre-intervention, Dur: during the intervention at 15 min, Post 0: immediately post-intervention, Post 15: at 15 min after the end of the stimulation
